# Effect of Melatonin and Calmodulin in an Idiopathic Scoliosis Model

**DOI:** 10.1155/2016/8460291

**Published:** 2016-11-30

**Authors:** Jun-Zhe Wu, Wen-Hua Wu, Li-Jiang He, Qing-Feng Ke, Long Huang, Zhang-Sheng Dai, Yu Chen

**Affiliations:** ^1^Department of Orthopaedics, The Second Affiliated Hospital, Fujian Medical University, Quanzhou, Fujian 362000, China; ^2^Department of Orthopaedics, Changhai Hospital, The Second Military Medical University, Shanghai 200433, China

## Abstract

*Background*. To explore influence of continuous illumination, luzindole, and Tamoxifen on incidence of scoliosis model of rats.* Methods*. Thirty-two one-month-old female rats were rendered into bipedal rats. The bipedal rats were divided into 4 groups: group A by intraperitoneal injection of luzindole and continuous illumination; group B by intraperitoneal injection of luzindole only; group C by intraperitoneal injection of luzindole and oral administration of Tamoxifen; and group D by intraperitoneal injection of equivalent saline. Radiographs were taken at 8th week and 16th week, and incidence and the Cobb angles of scoliosis were calculated. At 16th week, all rats were sacrificed. Before the sacrifice, the levels of calmodulin were measured in each group.* Results*. At 8th week, scoliosis occurred in groups A and B, with an incidence of 75% and 12.5%, respectively, while rats in group C or D had no scoliosis. At 16th week, scoliosis incidences in groups A and B were 57% and 62.5%, respectively. No scoliosis occurred in group C or D. Calmodulin in platelets in group B was significantly different, compared with groups A and D. There was no significant difference in calmodulin in platelets in groups B and C.* Conclusion*. By intraperitoneal injection of luzindole in bipedal rats, scoliosis rat models could be successfully made. Under light, incidence of scoliosis may be increased at an early period but it is reversible. Tamoxifen can suppress natural process of scoliosis.

## 1. Introduction

Currently, the main treatment options for idiopathic scoliosis (IS) consist of observation, brace, and surgical correction after it is found [1, 2]. The etiology and pathogenesis remain unknown. Considering that the spontaneous appearance of scoliosis in animals is rare, great efforts are required to achieve and develop scoliotic animal models. Growing interest has arisen in the past decades following the report of “idiopathic-like” scoliosis in animals with pinealectomy-induced melatonin deficiency. In 2009, we successfully made a scoliotic model in bipedal rats by using luzindole, a melatonin antagonist [3]. The objective of this study was to analyze whether the magnitude of curves of scoliosis may be influenced by 24-hour light exposure or the administration of pharmacological agents tamoxifen (TMX) in the luzindole-induced scoliosis model.

## 2. Information and Method

### 2.1. Experimental Rats

Before the experiment, protocol was approved by the local ethical committee. Thirty-two healthy SD female rats born at the same time were selected and were weaned and randomly divided into four groups, with eight rats in each after a week of adaptive growth. Forelegs and tails of rats in groups A, B, C, and D were amputated to make them as bipedal rats. Different groups were distinguished with picric acid. All experimental animals were purchased from the Animal Center of Fujian Medical University.

### 2.2. Experimental Materials

#### 2.2.1. Main Experimental Apparatus

Common operative instruments and items are as follows: disinfectants, normal saline, anhydrous alcohol, chloral hydrate anesthetic, needle holder, operating tweezers, operating scissors, 3-0 absorbable suture materials, aseptic dressing, aseptic hole towel, syringe, simple shadowless lamp, picric acid, continuous light source, small fan, and simple electronic balance.

#### 2.2.2. Main Experiment Reagents

1. Luzindole (antagonist for melatonin MEL-a receptor, 5 mg/single), Sigma (USA).
2. Tamoxifen (TMX) (receptor antagonist of calmodulin, 1 g/single), Sigma (USA).
3. Ficoll to separate and purify the blood platelets, Cedarlane, Ltd., number: CL5020-6.
4. CaM Rat Kit to measure the level of calmodulin in platelets of rats from each group, Wuhan Gene Biological Technology Co., Ltd.
5. Absolute alcohol, Hefei Sanding Chemical Co., Ltd.

### 2.3. Experimental Procedure

#### 2.3.1. Preparation of Rats and Cobb Angle Measurement

1. Radiograph taken before the experiment: all rates were one-month-old female. A full-length posteroanterior radiograph was taken for all rats, whose heads and bodies were straightened after intraperitoneal injection of anesthesia with 10% chloral hydrate (300 mg/kg) in the prone position. Photograph results revealed that experimental rats were all without scoliosis or other spine malformations, which met the experimental requirements.
2. Preparation of bipedal rats: with 10% chloral hydrate anesthesia (300 mg/kg), two forelegs in the scapulohumeral joint level and tails in basilar part were ligated with three unabsorbable sutures, the forelegs and tails were cut under the ligature, and broken ends were sutured to stop the bleeding. There was no obvious bleeding due to the firm ligation. After the operation, all bipedal rats were able to walk upright without difficulty in diet and life.
3. Experimental process: food and drinking water height were adjusted anytime according to the rat growth situation to make the bipedal rats walk. Operating time is 18:00 to 20:00 PM. All experimental rats were weighed every day. According to the weight of rats, the dose of luzindole for groups A, B, and C by intraperitoneal injection is 0.2 mg/kg, with equivalent normal saline by intraperitoneal injection for group D every day as a blank control. All-day lamination for group A with a light intensity of 100 lux was applied, and 10 mg/d of TMX was added to drinking water for group C. Body weight changes and the injected dose of luzindole or normal saline for rats in each group were recorded every day.
4. X-ray examination and Cobb angle measurements: At 8th and 16th week, full-length posteroanterior radiographs of the spine were taken with heads and bodies straightened after intraperitoneal injection of anesthesia with 10% chloral hydrate (300 mg/kg) in the prone position.
5. Before all rats were sacrificed at the end of the experiment, aortic blood was drawn from rats in each group and stored frozen to measure the concentration of calmodulin in platelets of rats in each group.

#### 2.3.2. Process of the Experiment and Life of Bipedal Rats

The room temperature was set between 20 and 24°C, with illumination from 8:00 to 19:00 for groups B, C, and D, full-day illumination for group A, and light intensity of 300 lux, supplied with adequate food and drinking water. Bipedal rats can walk normally on two feet, with slight anteflexion in the chest. In the process of foraging for food and water, the body can be completely upright. During the experimental process, the height of the food and drinking water was adjusted according to the growth rate of rats. Through deliberate training, rats were able to adapt to walking upright.

### 2.4. Statistical Analysis

SPSS19.0 software was used for statistical analysis of data. All data in the experiment were presented as average ± standard deviation. Two samples were analyzed by independent sample *t*-test, and multisamples were analyzed by one-way analysis of variance. *p* < 0.05 was considered statistically significant.

## 3. Results

### 3.1. Survival of Experimental Rats

A bipedal rat in group A suddenly died in the 3rd month of the experimental process. The cause of death was severe intestinal adhesion by dissection that led to intestinal obstruction.

### 3.2. Scoliosis of Experimental Rats

**Figure 1:**
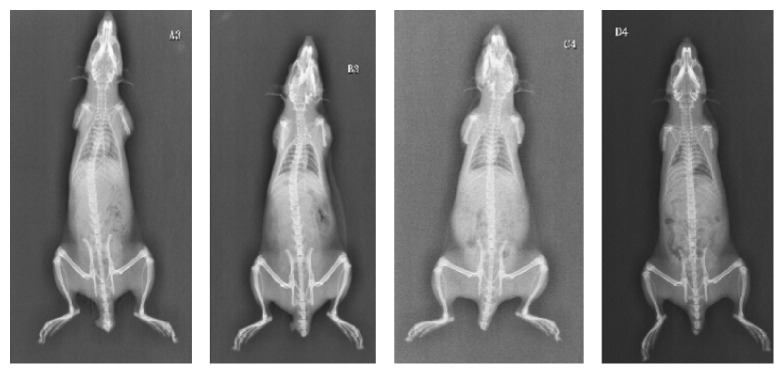
Radiograph taken for the bipedal rat in each group at 8th week.

**Figure 2:**
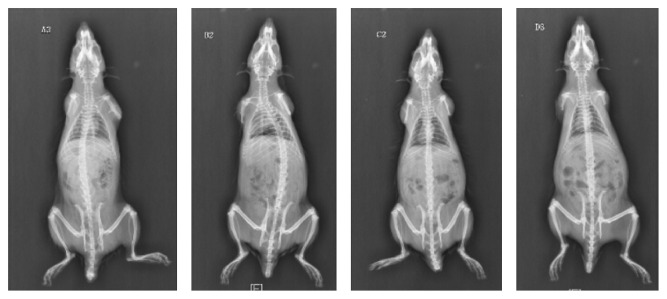
Radiograph taken for the bipedal rat in each group at 16th week.

(1) At 8th week, scoliosis occurred in groups A and B, with incidences of 75% and 12.5%, respectively. Scoliosis degrees of group A are between 10.8° and 16.8°, with an average of 12.4° ± 2.2°; and the average scoliosis in group B was 19.4°. The differences in scoliosis incidences between groups A and B are statistically significant (*p* = 0.041 < 0.05). Scoliosis did not occur in either group C or group D, with an incidence of zero. (2) At 16th week, scoliosis incidences for groups A and B were 57% and 62.5%, respectively; and the Cobb angles of scoliosis were between 10.1° and 17.9° for group A and between 18° and 30.3° for group B, with an average of 14.3° ± 3.3° and 25.2° ± 4.9°, respectively. No scoliosis occurred in either group C or group D, with an incidence of zero. The differences in incidences between groups A and B were not statistically significant (*p* = 1.000 > 0.05), while differences in incidences between groups B and C, as well as between groups B and D, were statistically significant (*p* = 0.026 < 0.05). The same *p* is because C and D groups have 0% of scoliosis (Tables 1 and 2) (Figures 1 and 2).

### 3.3. The Levels of Calmodulin in Platelets of Rats in Each Group

Table 3 shows the concentration of calmodulin in the platelets of rats in each group.

The level of calmodulin in platelets of experimental rats in group B was significantly lower than that in group D (control group), and the difference is statistically significant (*p* = 0.001 < 0.05). Group A has lower values compared to group B. The differences in calmodulin in platelets of rats between groups A and B were statistically significant (*p* = 0.001 < 0.05). There were no significant differences in calmodulin in platelets of rats between groups B and C (*p* = 0.998 > 0.05).

## 4. Discussion

### 4.1. Melatonin and Calmodulin

The association between melatonin and calmodulin in IS started from 1991. Benítez-King et al. revealed that part of the physiological function of melatonin was generated through CaM, and the association between melatonin and CaM is featured by high affinity and reversibility [4]. The possible mechanism of modulation is to adjust Ca2+ concentration within cells through G protein coupled receptor or through direct action on CaM [1]. However, in addition to cooperativity, Antón-Tay et al. found that melatonin may play the role of the antagonism against CaM by acting on hydrophobic Ca2+-dependent proteins within cells [5]. In patients with AIS, Machida et al. found that melatonin was lower and CaM in platelets was higher [2]. Research results of Agapito et al. revealed that Ca2+-CaM and calcium-activated neutral protease can adjust the synthesis of melatonin by adjusting the efflux of Ca2+ within the pineal body [6]. However, it can only affect the synthesis of melatonin at night and not in the day time [7]. Tamoxifen is known as kind of calmodulin antagonist which is currently used in cancer treatment based on their apoptotic effect by ways of anticalmodulin activity. Melatonin is known to be an apoptotic agent in cancer cells as well suggesting a similarity of action [8–10]. SERMs such as TMX and RLX may be effective in the reversal of osteopenia and, in parallel, the scoliotic deformity in animal models. In other words, the mechanism of action might not be solely calmodulin antagonism but also estrogen receptor modulation [11].

### 4.2. Fundamentals of the Experiment

Based on the above understanding, female bipedal rats were used in order to improve the success rate of modeling. Modeling time is a month, and the modeling method is through intraperitoneal injection of luzindole in bipedal rats, which was explored by members of the research group to ensure the occurrence of scoliosis in experimental rats. On this basis, through the addition of continuous illumination, oral calmodulin antagonists, and other factors, we can have a more in-depth understanding of the functions of those factors in the natural process of scoliosis by observing the occurrence of scoliosis, the extent of scoliosis, and changes of calmodulin. In addition, we can provide an animal model and experimental basis for the prevention and treatment of AIS.

### 4.3. The Experiment Condition

According to the AIS animal model research and research performed by scholars both at home and abroad, melatonin and calmodulin were detected to be abnormal [12, 13] both in animal models or in AIS patients. The researchers have also proposed the explanation for this mechanism [14–19], but most experiments was undertaken under the condition that melatonin reduced directly such as continuous illumination, pineal-body-removed chickens or pineal-body-removed rats, and mouse models of congenital absence of melatonin. We have applied reverse antagonism against receptors instead of reducing the quantity or quality of melatonin or calmodulin to supplement the positivity of the experiment and explain for the occurrence and mechanism of scoliosis to a certain degree.

### 4.4. The Incidence of Scoliosis in the Rats

During the experiments, based on the existence of MR (melatonin receptor) circadian rhythms proposed by Zhao et al. [20] such as peak value in the light phase (12:00), valley value in dark phrase (0:00), and the second peak value at dusk (18:00–20:00), the time of intraperitoneal injection of luzindole is controlled between 18:00 and 20:00. Compared to the incidence of scoliosis in bipedal rats reported by Wu et al. [21], the incidence in this study was lower. The reasons are the following: (1) two subtypes (MT1B and MT1A) exist in melatonin receptor 1, and the luzindole we use is MT1B high affinity receptor antagonist of melatonin. Therefore, MT1A low-affinity receptors were not antagonized. (2) According to the study by Zhao et al. [20], there are two peaks during melatonin secretion, in which we only suppress the first peak, and the second is not suppressed, which may be one of the reasons for the low incidence of scoliosis in our rat models. (3) Based on the experience of Akel et al. [22], after using calmodulin antagonists, scoliosis malformation was inhibited, which further decreased the incidence of scoliosis.

### 4.5. The Explanation for the Difference in Incidence of Scoliosis between Different Groups under Different Experimental Conditions

1. At 8th week, scoliosis incidences in rats in groups A and B were different, but there was no obvious difference in 16th week; and the scoliosis degrees of group B were more serious. Combined with relevant knowledge by Wu et al. [21], the reason may be the following: AIS occurs mainly around puberty, and the change in melatonin is just an inducing startup mechanism in the phase. When scoliosis malformation occurs, its further occurrence and development are caused mainly by the unbalanced force of spines and other factors. (1) In the early period for group A, with continuous illumination method tested by Ralph et al. [23], the amount of melatonin secreted in rats was significantly reduced. In addition to the antagonism of melatonin receptors, suppression and the sudden change in range of related functions of melatonin were higher than in group B, which makes its teratogenic effect occur earlier and makes it more significant. (2) When scoliosis malformation occurs, related mechanisms of melatonin no longer play their roles. As rats in group B are heavier than in group A, the unbalanced force of spines becomes more serious. Therefore, there was no obvious difference in scoliosis incidences in groups A and B, in addition to other unknown factors, while scoliosis malformation in group A was more serious than in group B [3].
2. The effect of TXM in group C: Akel et al. [11, 22] in their study concluded that calmodulin antagonists can suppress the occurrence and development of scoliosis in rats because of lack of melatonin. In our study in which we introduced the method of luzindole the interfering condition of TMX and final results were similar to the result obtained by Akel et al. In the whole course of the experiment, scoliosis malformation did not occur in rats in group C. As a result, we believe that calmodulin antagonists can suppress the occurrence and development of scoliosis in animal models caused by melatonin receptor antagonists.

### 4.6. Explanation of the Different Levels of Calmodulin in Platelets of Each Group

1. Calmodulin in platelets of rats in groups B and D was significantly different. Through the hint and knowledge that the intraperitoneal injection of luzindole can reduce calmodulin in platelets and in patients with AIS and that reducing melatonin can increase this (as proposed by Machida et al.), we believe that intraperitoneal injection of luzindole not only reduces melatonin in rats but also (1) promotes the secretion of melatonin in rats through the negative feedback regulatory mechanism and, (2) for the suppression of MT1B receptors, may increase the sensitivity of MT1A and MT2, further reducing calmodulin in platelets through some factors. Thus, we believe that a lack or loss of function of melatonin is the prerequisite for scoliosis to occur, and melatonin antagonists can also make calmodulin in platelets decrease significantly.
2. Calmodulin in platelets of rats in groups B and C was not significantly different. A hint is that calmodulin antagonists do not directly make a significant change in calmodulin in platelets but play role of suppressing the occurrence and development of scoliosis by the loss of function of calmodulin in platelets. Combined with a previous research, we believe that calmodulin is also necessary for scoliosis, but not a dominant factor. The teratogenic effects only relate to the existence of functions of calmodulin.
3. Calmodulin in platelets in rats in groups A and B was significantly different. It suggests that light can make calmodulin in platelets significantly lower. We know that light can make melatonin significantly lower and reducing melatonin may increase calmodulin [2], while calmodulin in platelets in group A was significantly lower than in group B. Therefore, light may, through other unknown mechanisms, reduce the level of calmodulin in platelets.

## Figures and Tables

**Table 1:** Scoliosis of experimental rats in each group.

Group	Occurrence of scoliosis	
	8th week	16th week
Group A	6/8	4/7
Group B	1/8	5/8
Group C	0/8	0/8
Group D	0/8	0/8

**Table 2:** The Cobb angles in bipedal rats in Groups A and B.

Group	Number of weeks	
	8th week	16th week
A	12.4° ± 2.2°	14.3° ± 3.3°
B	19.4° ± 0°	25.2° ± 4.9°

**Table 3:** Calmodulin in platelets of rats in each group: unit (ng/L).

Group	Mean	Standard deviation
Group A	87.6	9.4
Group B	121.6	3.7
Group C	120.5	5.1
Group D	152	3.8
